# Hospital at home digital twin for the management of patients with frailty: a scoping review protocol

**DOI:** 10.1136/bmjopen-2024-093418

**Published:** 2025-06-17

**Authors:** Faiza Yahya, Matthew Cooper, Mohamad Kassem, Hamde Nazar

**Affiliations:** 1 Newcastle Patient Safety Research Collaboration, Newcastle University, Newcastle upon Tyne, UK; 2 School of Engineering, Newcastle University, Newcastle upon Tyne, UK

**Keywords:** Frailty, Digital Technology, Patient Care Management

## Abstract

**Abstract:**

**Introduction:**

Patients with frailty are at risk of adverse outcomes such as mortality, falls, deconditioning and hospital readmissions. With an increasingly ageing population and a greater likelihood of frailty, there is a significant need to ensure that patients are managed in the right place and at the right time. There has been a focus on offering hospital-level care at home as a way to meet this need, incorporating strategies to integrate care and use digital solutions. Digital twin (DT) technology is one advancement, offering a virtual replica of an object/environment, which has the potential to make use of real-time data personalised for an individual patient and/or setting to inform and support patient management decisions. We are yet to realise the full potential of this new way of integrated working and technological advancements. This scoping review aims to ascertain the current evidence for the components of the DT architecture to enable the monitoring and management of patients with frailty living at home.

**Methods:**

This scoping review will follow the Joanna Briggs Institute methodology for scoping reviews and will be reported following the Preferred Reporting Items for Systematic Reviews Extension for Scoping Reviews guidelines. The following electronic databases will be searched: Medline, Embase, CINAHL, Cochrane CENTRAL, Web of Science and Scopus. Relevant websites will be searched for grey literature or case reports to capture the required information, as well as any documents provided by stakeholders. Primary studies, published in the English language from 2019 to the present day, which report on the monitoring or management of patients with long-term conditions and frailty within their home environment, will be included. Screening will be conducted by at least two independent reviewers against eligibility criteria, and a piloted data extraction form will be used to align with the research questions. Qualitative content analysis will be used. Data will be presented in tabular form, as well as descriptive and illustrative formats, to address the objectives of this review.

**Ethics and dissemination:**

This scoping review does not require ethical approval. The findings of this review will be disseminated through peer-reviewed journals and conferences and will support the development of a conceptual model of a hospital-at-home DT for the management of patients with frailty.

**STRENGTHS AND LIMITATIONS OF THIS STUDY**

- This scoping review will examine the components to support digital twin (DT) architecture necessary for the monitoring of patients with frailty, at home, adopting learning to identify what is needed to build a DT architecture from other industries.
- The growth in technology and machine intelligence, however, is rapidly advancing, and therefore, the proposed review is limited by the availability of evidence. To mitigate this, the review question is broad, ensuring any data addressing frailty in the patient’s home is captured.
- This review will be the first review to map the plausible architecture for DT models in healthcare for patients with frailty, providing a blueprint for future development of agents and systems.

## Introduction

 Patients with frailty are at high risk of adverse outcomes such as mortality,^1^ hospital admissions,^1 2^ falls and admission to long-term care.^3^ Frailty is more prevalent with older adults and is associated with higher rates of comorbidities and disability.^2^ Frailty can often be viewed as an age-related clinical condition with a decline in physiological capacity.^3^ However, it is important to recognise that frailty can also affect younger individuals with multimorbidities at increased risk of mortality.^4^ The UK has an increasingly ageing population compounding pressures on the healthcare system. Therefore, being able to recognise and manage frailty becomes fundamental to meeting often complex health and social care requirements and preventing harm. Patients with frailty may be vulnerable at times of stress such as acute illness or changes in circumstances. Frailty status and severity are also dynamic and can decline or improve over time.^3 5^ This can affect the management of treatment decisions, management plans and recovery expectations.^3^ Hence, management relies on interventions that are personalised to the patient and their individual circumstances at the time, for example, within their home environment.

Strategies to recognise, prevent and manage frailty and deterioration in individuals have grown over the years, but there remain limitations in the evidence base,^3 5^ and it is not clear how patients with frailty are most effectively managed in their own home. The definitions of frailty over the years have often been difficult to determine. However, for this review, we are using the British Geriatric Society definition as “a distinctive health state related to the ageing process in which multiple body systems gradually lose their in-built reserves”^6 7^ or if the included studies have used frailty in defining the population studied.

The management of patients with frailty in their own home presents challenges and implications compared with hospital settings, and an emphasis on proactive care is encouraged. Studies have shown that implementing effective interventions for preventing frailty can improve the quality of life of older adults, maintain their independence and reduce costs on the health and social care systems.^2 8 9^

Over recent years, there has been an increasing drive to achieve integrated healthcare for an ageing population and ensure patients receive care in the right place at the right time. This can mean ensuring more access to care in the community. Such measures include hospital-at-home (HaH) models, which deliver hospital-level care to patients who are acutely unwell in their own home, rather than being in hospital.^10^ These can be classified as either of two types of models: admissions avoidance (step-up) and/or to facilitate early discharge (step-down). HaH can be a very convenient and acceptable model of care for patients with frailty.^11^ These rapidly evolving models of care rely on multidisciplinary teams, digital technology and intuitive systems to deliver their care, as well as good communication and coordination.^12^ However, implementation has been varied and evidence is inconsistent or lacking in many aspects.^12 13^ These models involve new ways of working, adaptability and providing care in less clinically controlled environments, such as a hospital ward, where different risks need to be proactively monitored. Within the home setting, there is a more significant need for effective risk assessment, status prediction and care management than in a typical clinical setting to support safe and effective patient care.

Currently, there is a focus to standardise these models; however, it has also been argued that flexibility is required at local levels to meet local population needs.^14^ Conceptually, HaH models should consist of a generalisable structure with key operational aspects and components, using patient-level data to inform care delivery. However, there is currently a lack of evidence about which patient-level data to measure, the mechanism of measurement and how these data most effectively inform care decisions.

Digital twin (DT) technology offers the opportunity to create a virtual clone of a physical object/environment/situation, which uses real-time information to replicate its existing state and can be used to forecast future possibilities.^15^ DT models are commonly used in the aviation and manufacturing industries.^16^ However, little is known about their application in healthcare systems. Smart healthcare monitoring systems are increasingly being implemented in patient care. This real-time data can potentially be captured to allow care providers to monitor patients’ condition(s) and behaviours within the architecture of a DT to predict future outcomes, trigger/inform care interventions and reduce harm. Within the context of HaH, DT will facilitate a real-time opportunity for patients to live well in their homes, optimising care and triggering escalation for medical and social care interventions, leading to reduced harm.

**Figure 1:**
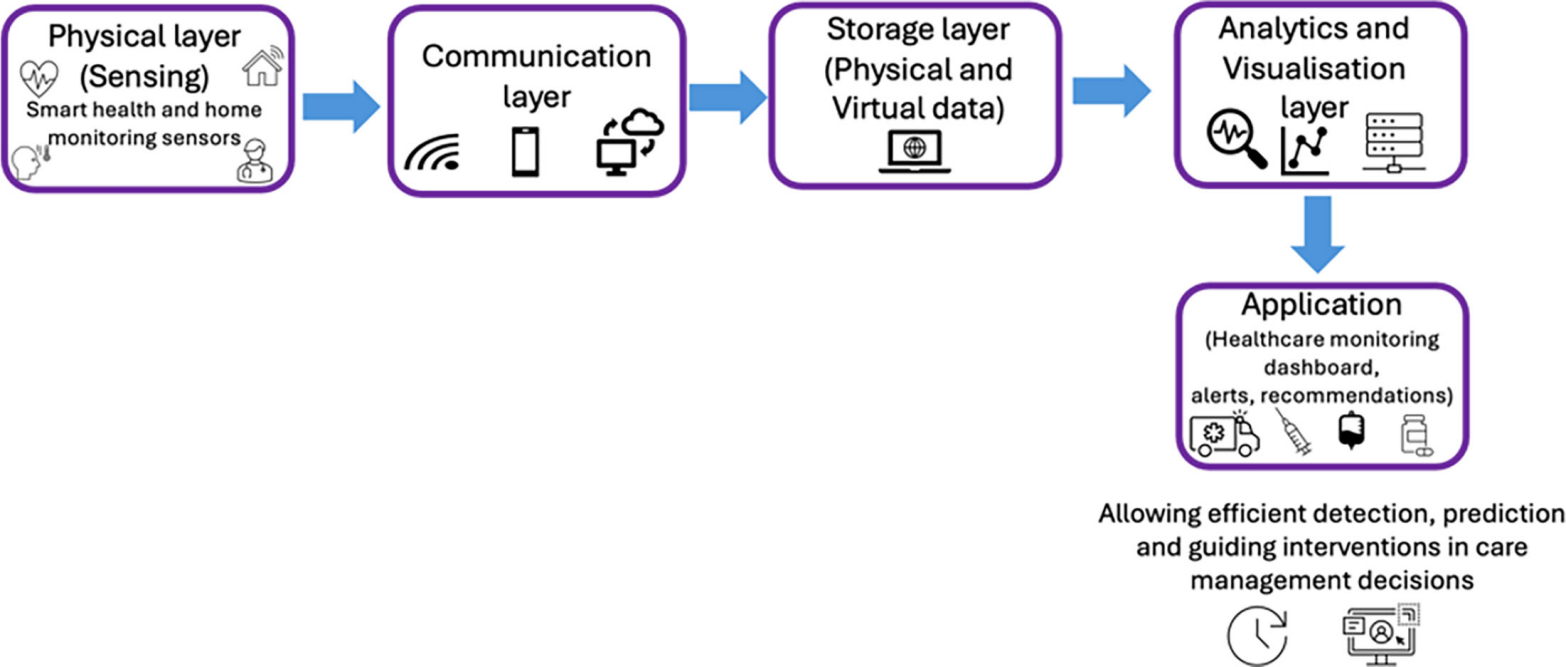
Simplified paradigm for healthcare digital twin architecture. Adapted from Al-Ali *et al*.^16^

Various DT applications have been explored with limited development within health and social care.^16^ DT models have been conceptualised through a multiple-layered architecture (see figure 1) where information is needed about the physical layer (sensing), form/mode of communication, the virtual space (including data storage, data analytics and visualisation) and the application layer.^16^ The construction of a healthcare DT takes into consideration multiple systems and stages. Information may be collected from wearable mobile sensors, smart health monitoring or smart home monitoring sensors. This dynamically updated real-time information needs to be securely transferred (eg, via a secure cloud platform). This information is then stored in a virtual space and analysed using data simulation, modelling and machine learning to enable analysis and visualisation of potential outcomes that can inform health and care decisions. Examples of where DT has been used to support patient care using precision medicine are seen in cardiology, surgery and orthopaedics.^17^ They have also been tested in the context of supporting diabetes management.^15 18^ However, beyond prototyping, evidence supporting the use of DT within a real-life context is limited.

This scoping review aims to capture the evidence about the multiple layers of a DT architecture that exist within the current literature that would support the HaH model of care for patients with frailty. A preliminary search on MEDLINE identified that the evidence is sparse in the area of HaH models and negligible in adopting the conceptualisation of DT. Therefore, this review will investigate evidence across the monitoring and management of patients with frailty in the home setting and will aim to capture information about any of the DT architectural layers that are included and discussed. A scoping review approach was identified as the ideal method to capture the required information from a wide range of sources. Scoping reviews are widely used as a systematic, rigorous approach to knowledge synthesis to map key concepts and the existing empirical evidence in an area that has not been extensively reviewed.^19^ Furthermore, no systematic reviews or scoping reviews were identified to answer this research question in the context of HaH, and the evidence that exists is variable. Literature reviews have shown that healthcare DTs are growing out of their infancy phase, but there remains a paucity of evidence on whole body or hospital systems.^20^ Emerging evidence^21^ in the design and implementation of DTs for use in the home shows great potential; however, this systematic review does not capture any literature relevant to HaH or acute care. Furthermore, our study aims to explore DT-enabling tools not just DTs in the home, which would advance our understanding and describe the granularity of the architecture according to the five layers shown in figure 1. This aims to advance our understanding and progression for developing a useful blueprint for a HaH DT. Additionally, unlike other reviews, we aim to include qualitative studies to enhance our understanding of the acceptability and usability of DT-enabling tools. This review will propose one conceptual model for HaH DT, rather than multiple conceptual models for different applications, which would be more useful to clinical practitioners for a holistic healthcare application.

## Methods and analysis

The proposed scoping review will be conducted following the Joanna Briggs Institute (JBI) methodology for scoping reviews,^22^ based on the original Arksey and O’Malley methodology.^23^

The population, concept and context mnemonic was used to develop the research question, search and inclusion criteria as recommended by the JBI guidelines for scoping reviews^22^ (see table 1).

**Table 1:** Inclusion criteria

Population	Patients with long-term conditions and frailty
Concept	Studies involving the use of tools used to support the monitoring and management of patients with frailty in the home setting.
Context	Studies investigating any care provided in the home setting or transfer to and from a patients’ home.

### Aim

To ascertain the evidence for the components of the DT architecture necessary for the monitoring and management of patients with frailty at home.

The following subquestions will be used as a framework to address the overall research aim and will support the collection of data pertaining to the architectural layers of DT; however, each study will not need to and may not answer all subquestions.

### Subquestions

- What tools are being used to identify patient risk, monitor disease state and/or well-being and support decision-making in home-based care for patients with frailty and the measurement of effectiveness?
- How is this information being collected, recorded and transmitted and by what mechanism? (sensing, communication and storage)
- What outcomes are being evaluated and how are the results being used to inform patient care? (analytics/decision-making, visualisation/presentation)
- Have there been any challenges or opportunities identified with these tools to support safer monitoring and management of patients with frailty?

### Types of sources

Primary studies, published in the English language, which report on the monitoring or management of patients with long-term conditions and frailty within their own home environment will be included. Studies will not be limited by geographical location. Published evidence from 2019 to the present day will be included to incorporate the most relevant and up-to-date information. Furthermore, it is recognised that since the COVID-19 pandemic, the use of digital technology, remote monitoring and the roll-out of HaH models have vastly increased and advanced since previous models of care, and we wish to capture the most relevant information. Additionally, recent studies have highlighted the lack of studies on DTs prior to 2019.

Studies may include qualitative, quantitative and mixed-method studies, either observational, experimental or quasiexperimental. This can include randomised controlled trials, non-randomised controlled trials, before and after studies, cohort studies, case–control studies, cross-sectional studies, evaluation reports, case reports and grey literature.

The studies must include the reporting of outcomes related to effectiveness, usability, acceptability and safety.

### Exclusion criteria

Review articles, protocols and conference abstracts will be excluded as they will not include the primary data required to meet the review objectives.

### Search strategy

The following electronic databases will be searched: Medline, Embase, CINAHL, Cochrane CENTRAL, Web of Science and Scopus. The FutureNHS Platform for localised evaluation reports will also be searched, as well as NHS England websites, Department of Health websites, the Hospital at Home Society website and related links.

Forward citation searching from included papers, snowballing and contacting experts in the field may also be adopted for comprehensiveness. The search will be limited to English language only and publications between 2019 and present.

As recommended by the JBI guidelines, a three-step search strategy will be adopted.^19^ An initial limited search of two databases (Medline and Embase), followed by an analysis of keywords to refine the search, will be implemented. This refined search strategy will be used and adapted for a search across all databases. Third, the reference lists of identified full-text sources will be searched for comprehensiveness. Support from a research librarian has been sought in the formulation of the search terms (see table 2 and online supplemental appendix 1).

**Table 2:** Search terms

PCC	Theme	Synonyms/keywords
Population	Patients with frailty	frail elderly, frailty, frail*
Concept	Monitoring or management	“Home monitoring”, health care management, “care decisions”, “risk assessment”, “health* monitoring”, patient monitoring, telemonitoring, telecare, telemedicine, Patient specific model*ing, digital monitoring, remote sensing, digital*, digital twin
Context	Home setting	Hospital adj2 at home, “Virtual Ward”, Home, community dwelling person, independent living,“acute care at home”, “hospital in the home”, “home hospital*”, “hospital-based home care”, “rapid response”, home care, domiciliary

PCC, population, concept and context.

### Source of evidence selection

All results retrieved from the databases will be imported into Rayyan for screening. Any grey literature such as case studies or evaluation studies that cannot be imported into Rayyan will be added to an Excel spreadsheet for screening. For any literature that cannot be retrieved, the authors of the papers will be contacted to request this. Articles will be reviewed by more than one author for validation. A pilot stage will be conducted where two or more authors will review a sample of 25 titles/abstracts, and any conflicts or discrepancies will be resolved through discussion. Agreement must reach 75% or greater for the selection of articles to continue.^22^ Reporting of all studies will follow the Preferred Reporting Items for Systematic Reviews and Meta-Analyses extension for Scoping Reviews.^24^

### Data extraction and charting

A data extraction tool will be used, and a draft is provided in online supplemental appendix 2. This has been developed by the authors and piloted on a small number of studies from a pilot search. A pilot will be conducted and discussed among authors once the full search has taken place, and the data extraction tool will be modified as necessary to meet the objectives.

### Analysis of the evidence

The results extracted from the data charting process will be mapped in tabular format in relation to the objectives of this review. A qualitative content analysis process^25^ will be used, involving both inductive and deductive approaches. This will allow results to be systematically presented numerically, descriptively and reveal key concepts. Where there is quantitative data, this will be analysed by narrative synthesis to support the evidence.

#### Presentation of the results

Results will be presented as a combination of tabular forms and charts, depending on the nature of the results. Descriptive information (ie, characteristics of the studies) will be presented in tabular form, and other findings that answer the research questions will be presented as a visual chart format that best represents the findings and will inform an illustrative conceptual model.

#### Critical appraisal

Critical appraisal is not required in line with JBI guidelines for scoping reviews.^22^

#### Patient and public involvement (PPI) statement

This protocol was developed without PPI, however, the findings from the scoping review will inform future research that will require subsequent PPI.

## Implications for practice

The identification of the key architecture needed to build a DT for the management of patients with frailty in their homes will be presented as a conceptual model. This model can be validated in collaboration with experts from the digital technology industry and the health and social care sector to advance the evidence base for an innovative, technologically supported model of care for patients in their own homes. This review has focused on patients with frailty; however, the conceptual model and method of construction will be important, potentially generalisable to and replicable for wider patient groups (eg, those experiencing heart failure) and in various environments of residence (eg, care homes) and/or environments of care (eg, hospices). The conceptual model will provide the blueprint for building a DT prototype that can be rigorously tested in patient populations for effectiveness and cost-effectiveness. It is acknowledged that process evaluations will be vital alongside such experimental studies to investigate aspects of implementation, wider adoption and scale-up.

## Supplementary material

10.1136/bmjopen-2024-093418 online supplemental file 1

10.1136/bmjopen-2024-093418 online supplemental file 2

10.1136/bmjopen-2024-093418 online supplemental file 3

## References

[1] Hao Q, Zhou L, Dong B (2019). The role of frailty in predicting mortality and readmission in older adults in acute care wards: a prospective study. Sci Rep.

[2] Fried LP, Tangen CM, Walston J (2001). Frailty in older adults: evidence for a phenotype. J Gerontol A Biol Sci Med Sci.

[3] Dent E, Martin FC, Bergman H (2019). Management of frailty: opportunities, challenges, and future directions. The Lancet.

[4] Hanlon P, Nicholl BI, Jani BD (2018). Frailty and pre-frailty in middle-aged and older adults and its association with multimorbidity and mortality: a prospective analysis of 493 737 UK Biobank participants. Lancet Public Health.

[5] Gill TM, Gahbauer EA, Allore HG (2006). Transitions between frailty states among community-living older persons. Arch Intern Med.

[6] British Geriatric Society (2014). Introduction to frailty, fit for frailty part 1. https://www.bgs.org.uk/resources/introduction-to-frailty.

[7] British Geriatric Society (2018). Frailty: what’s it all about?. https://www.bgs.org.uk/resources/frailty-what%E2%80%99s-it-all-about.

[8] Walters K, Frost R, Kharicha K (2017). Home-based health promotion for older people with mild frailty: the HomeHealth intervention development and feasibility RCT. Health Technol Assess.

[9] Muszalik M, Kotarba A, Borowiak E (2021). Socio-Demographic, Clinical and Psychological Profile of Frailty Patients Living in the Home Environment and Nursing Homes: A Cross-Sectional Study. Front Psychiatry.

[10] Hospital at Home Society (2023). What is hospital at home?. https://www.hospitalathome.org.uk/whatis.

[11] Westby M, Ijaz S, Savović J (2024). Virtual wards for people with frailty: what works, for whom, how and why-a rapid realist review. Age Ageing.

[12] Westby M, Ijaz S, Savović J Rapid realist review of virtual wards for people with frailty. *Geriatric Medicine*.

[13] Norman G, Bennett P, Vardy ERLC (2023). Virtual wards: a rapid evidence synthesis and implications for the care of older people. Age Ageing.

[14] Hakim R (2023). Realising the potential of virtual wards. https://www.nhsconfed.org/publications/realising-potential-virtual-wards.

[15] Dhanaraj RK, Murugesan S, Balusamy B (2022). Digital twin technologies for healthcare 4.0.

[16] Al-Ali AR, Gupta R, Zaman Batool T (2020). Digital twin conceptual model within the context of internet of things. Future Internet.

[17] Sun T, He X, Li Z (2023). Digital twin in healthcare: Recent updates and challenges. Digit Health.

[18] Chu Y, Li S, Tang J (2023). The potential of the Medical Digital Twin in diabetes management: a review. Front Med (Lausanne).

[19] Levac D, Colquhoun H, O’Brien KK (2010). Scoping studies: advancing the methodology. Implement Sci.

[20] Machado TM, Berssaneti FT (2023). Literature review of digital twin in healthcare. Heliyon.

[21] Zafar RO, Rybarczyk Y, Borg J (2024). A systematic review of digital twin technology for home care. ACM Trans Comput Healthcare.

[22] Peters M, Godfrey C, McInerney P (2020). JBI manual for evidence synthesis.

[23] Arksey H, O’Malley L (2005). Scoping studies: towards a methodological framework. Int J Soc Res Methodol.

[24] Tricco AC, Lillie E, Zarin W (2018). PRISMA Extension for Scoping Reviews (PRISMA-ScR): Checklist and Explanation. Ann Intern Med.

[25] Elo S, Kyngäs H (2008). The qualitative content analysis process. J Adv Nurs.

