# Supplementary material for: Hospital at home digital twin for the management of patients with frailty: a scoping review protocol
Source: BMJ Open. 2025 Jun 17;15(6):e093418. doi: 10.1136/bmjopen-2024-093418 (PMC12182040; doi:10.1136/bmjopen-2024-093418)
Supplement: online supplemental file 1 [file bmjopen-15-6-s001.docx]

**Searches for Scoping review –**

**Hospital at home digital twin for the management of patients with frailty**

**Ran 11.9.24**

**Embase <1974 to 2024 September 10>**

1 frail elderly/ or frailty/ 43608

2 frail*.ti,ab,kf. 63825

3 1 or 2 71263

4 "home monitoring".ti,ab,kf. 4018

5 health care management.ti,ab,kf. 1805

6 "care decision*".ti,ab,kf. 6873

7 risk assessment/ 774227

8 "health* monitoring".ti,ab,kf. 9240

9 patient monitoring.ti,ab,kf. 7684

10 telemonitoring/ 6543

11 telemedicine/ or telecare/ 51272

12 "patient specific model*ing".ti,ab,kf. 635

13 "digital* monitor*".ti,ab,kf. 479

14 remote sensing/ 17541

15 digital*.ti,ab,kf. 283967

16 digital twin/ 948

17 4 or 5 or 6 or 7 or 8 or 9 or 10 or 11 or 12 or 13 or 14 or 15 or 16 1143778

18 (Hospital adj2 at home).ti,ab,kf. 2359

19 "virtual ward".ti,ab,kf. 258

20 home.ti,ab,kf. 430755

21 community dwelling person/ 12574

22 independent living/ 8390

23 "acute care at home".ti,ab,kf. 58

24 "hospital in the home".ti,ab,kf. 380

25 "home hospital*".ti,ab,kf. 1061

26 "hospital-based home care".ti,ab,kf. 187

27 "rapid response".ti,ab,kf. 12354

28 home care/ 73255

29 domiciliary.ti,ab,kf. 3975

30 18 or 19 or 20 or 21 or 22 or 23 or 24 or 25 or 26 or 27 or 28 or 29 484082

31 3 and 17 and 30 1091

32 limit 31 to (english language and yr="2019 -Current") **678**

**Ovid MEDLINE(R) ALL <1946 to September 10, 2024>**

1 Frail Elderly/ or Frailty/ 23084

2 frail*.ti,ab,kf. 41509

3 1 or 2 46143

4 "home monitoring".ti,ab,kf. 2525

5 health care management.ti,ab,kf. 1498

6 "care decision*".ti,ab,kf. 5312

7 Risk Assessment/ 316976

8 "health* monitoring".ti,ab,kf. 9082

9 patient monitoring.ti,ab,kf. 5307

10 telecare.mp. 1035

11 Telemedicine/ or telemonitoring.mp. 42878

12 "patient specific model*ing".ti,ab,kf. 526

13 "digital* monitor*".ti,ab,kf. 327

14 Remote Sensing Technology/ 4411

15 digital*.ti,ab,kf. 229480

16 Digital twin.mp. 1055

17 4 or 5 or 6 or 7 or 8 or 9 or 10 or 11 or 12 or 13 or 14 or 15 or 16 608037

18 (Hospital adj2 at home).ti,ab,kf. 1567

19 "virtual ward".ti,ab,kf. 108

20 home.ti,ab,kf. 300300

21 Independent Living/ or community dwelling person.mp. 12883

22 "acute care at home".ti,ab,kf. 20

23 "hospital in the home".ti,ab,kf. 230

24 "home hospital*".ti,ab,kf. 672

25 "hospital-based home care".ti,ab,kf. 163

26 "rapid response".ti,ab,kf. 9632

27 Home Care Services/ 37290

28 domiciliary.ti,ab,kf. 2960

29 18 or 19 or 20 or 21 or 22 or 23 or 24 or 25 or 26 or 27 or 28 332401

30 3 and 17 and 29 521

31 limit 30 to (english language and yr="2019 -Current") **231**

**CINAHL (EBSCOhost)**

**Print Search History**

| Wed, September 11, 2024 03:47:53 pm   \| # \| Query \| Limiters/Expanders \| Results \| \| --- \| --- \| --- \| --- \| \| S29 \| S1 AND S27 AND S28 \| Limiters - Publication Date: 20190101-20241231 Expanders - Apply equivalent subjects Narrow by Language: - english Search modes - Proximity \| 439 \| \| S28 \| S14 OR S15 OR S16 OR S17 OR S18 OR S19 OR S20 OR S21 OR S22 OR S23 OR S24 OR S25 OR S26 \| Expanders - Apply equivalent subjects Search modes - Proximity \| 174,808 \| \| S27 \| S2 OR S3 OR S4 OR S5 OR S6 OR S7 OR S8 OR S9 OR S10 OR S11 OR S12 OR S13 \| Expanders - Apply equivalent subjects Search modes - Proximity \| 278,652 \| \| S26 \| AB domiciliary \| Expanders - Apply equivalent subjects Search modes - Proximity \| 662 \| \| S25 \| (MH "Home Health Care") OR "home care services" \| Expanders - Apply equivalent subjects Search modes - Proximity \| 26,848 \| \| S24 \| AB home care services \| Expanders - Apply equivalent subjects Search modes - Proximity \| 3,715 \| \| S23 \| AB "rapid response" \| Expanders - Apply equivalent subjects Search modes - Proximity \| 1,785 \| \| S22 \| AB "rapid response" \| Expanders - Apply equivalent subjects Search modes - Proximity \| 1,785 \| \| S21 \| AB "hospital-based home care" \| Expanders - Apply equivalent subjects Search modes - Proximity \| 63 \| \| S20 \| AB "home hospital*" \| Expanders - Apply equivalent subjects Search modes - Proximity \| 225 \| \| S19 \| AB "acute care at home" \| Expanders - Apply equivalent subjects Search modes - Proximity \| 55 \| \| S18 \| AB "hospital in the home" \| Expanders - Apply equivalent subjects Search modes - Proximity \| 101 \| \| S17 \| "community dwelling" OR (MH "Home Environment") \| Expanders - Apply equivalent subjects Search modes - Proximity \| 33,639 \| \| S16 \| AB home \| Expanders - Apply equivalent subjects Search modes - Proximity \| 132,332 \| \| S15 \| AB virtual ward \| Expanders - Apply equivalent subjects Search modes - Proximity \| 79 \| \| S14 \| AB hospital n2 at home \| Expanders - Apply equivalent subjects Search modes - Proximity \| 8,686 \| \| S13 \| TX digital twin* \| Expanders - Apply equivalent subjects Search modes - Proximity \| 135 \| \| S12 \| AB digital* \| Expanders - Apply equivalent subjects Search modes - Proximity \| 41,698 \| \| S11 \| AB remote sensing \| Expanders - Apply equivalent subjects Search modes - Proximity \| 199 \| \| S10 \| AB digital* monitor* \| Expanders - Apply equivalent subjects Search modes - Proximity \| 540 \| \| S9 \| AB patient specific model*ing \| Expanders - Apply equivalent subjects Search modes - Proximity \| 88 \| \| S8 \| SU ( telecare or telemedicine or telehealth ) OR SU telemonitoring \| Expanders - Apply equivalent subjects Search modes - Proximity \| 32,521 \| \| S7 \| AB patient monitoring \| Expanders - Apply equivalent subjects Search modes - Proximity \| 15,135 \| \| S6 \| AB "health* monitoring" \| Expanders - Apply equivalent subjects Search modes - Proximity \| 769 \| \| S5 \| (MH "Risk Assessment") \| Expanders - Apply equivalent subjects Search modes - Proximity \| 171,751 \| \| S4 \| AB care decision* \| Expanders - Apply equivalent subjects Search modes - Proximity \| 13,828 \| \| S3 \| AB health care management \| Expanders - Apply equivalent subjects Search modes - Proximity \| 7,786 \| \| S2 \| AB home monitoring \| Expanders - Apply equivalent subjects Search modes - Proximity \| 2,004 \| \| S1 \| (MM "Frail Elderly") OR ( frailty or frail* ) \| Expanders - Apply equivalent subjects Search modes - Proximity \| 23,558 \|   **Cochrane CENTRAL**  Search Name: SCR HaH DT  Date Run: 12/09/2024 11:21:58  Comment:  ID Search Hits  #1 MeSH descriptor: [Frailty] explode all trees 814  #2 MeSH descriptor: [Frail Elderly] explode all trees 1149  #3 frail* 6684  #4 #1 OR #2 OR #3 6684  #5 ("home monitoring"):ti,ab,kw OR ("health care management"):ti,ab,kw OR (care decision*):ti,ab,kw OR (health* monitoring):ti,ab,kw OR ("patient monitoring"):ti,ab,kw (Word variations have been searched) 63782  #6 MeSH descriptor: [Risk Assessment] explode all trees 13686  #7 MeSH descriptor: [Telemedicine] explode all trees 4989  #8 (telemonitoring):ti,ab,kw (Word variations have been searched) 1510  #9 (telecare):ti,ab,kw (Word variations have been searched) 287  #10 MeSH descriptor: [Remote Sensing Technology] explode all trees 75  #11 (digital*):ti,ab,kw (Word variations have been searched) 23148  #12 (digital twin):ti,ab,kw (Word variations have been searched) 88  #13 #5 OR #6 OR #7 OR #8 OR #9 OR #10 OR #11 OR #12 101470  #14 (hospital NEXT at home):ti,ab,kw (Word variations have been searched) 724  #15 ("virtual ward"):ti,ab,kw (Word variations have been searched) 11  #16 (home):ti,ab,kw (Word variations have been searched) 65276  #17 MeSH descriptor: [Independent Living] explode all trees 1177  #18 ("acute care at home"):ti,ab,kw (Word variations have been searched) 10  #19 ("hospital in the home"):ti,ab,kw (Word variations have been searched) 50  #20 (home NEXT hospital*):ti,ab,kw (Word variations have been searched) 173  #21 ("hospital-based home care"):ti,ab,kw (Word variations have been searched) 22  #22 ("rapid response"):ti,ab,kw (Word variations have been searched) 515  #23 MeSH descriptor: [Home Care Services] explode all trees 3132  #24 ("domiciliary"):ti,ab,kw (Word variations have been searched) 564  #25 #14 OR #15 OR #16 OR #17 OR #18 OR #19 OR #20 OR #21 OR #22 OR #23 OR #24 66968  #26 #4 AND #13 AND #25 with Cochrane Library publication date Between Jan 2019 and Sep 2024 **207** |
| --- | --- | --- | --- | --- | --- | --- | --- | --- | --- | --- | --- | --- | --- | --- | --- | --- | --- | --- | --- | --- | --- | --- | --- | --- | --- | --- | --- | --- | --- | --- | --- | --- | --- | --- | --- | --- | --- | --- | --- | --- | --- | --- | --- | --- | --- | --- | --- | --- | --- | --- | --- | --- | --- | --- | --- | --- | --- | --- | --- | --- | --- | --- | --- | --- | --- | --- | --- | --- | --- | --- | --- | --- | --- | --- | --- | --- | --- | --- | --- | --- | --- | --- | --- | --- | --- | --- | --- | --- | --- | --- | --- | --- | --- | --- | --- | --- | --- | --- | --- | --- | --- | --- | --- | --- | --- | --- | --- | --- | --- | --- | --- | --- | --- | --- | --- | --- | --- | --- | --- | --- |

Cochrane reviews = 4

Trials = **203**

**Web of Science**

(ALL=(frailty OR frail elderly)) OR TS=(frail*)

AND

TS=((“Home monitoring” OR “health care management” OR “care decisions” OR “health* monitoring” OR “patient monitoring” OR “telemonitoring” OR “telecare” OR “telemedicine” OR “Patient specific model*ing" OR “digital monitoring” OR “remote sensing” OR “digital*” OR “digital twin”))

AND

TS=((hospital NEAR/2 at home OR “virtual ward” OR Home OR “community dwelling person” OR independent living OR “acute care at home” OR “hospital in the home” OR “home hospital*” OR “hospital-based home care” OR “rapid response” OR home care OR domiciliary))

Results after limits 2019-2024 and English = **164**

**
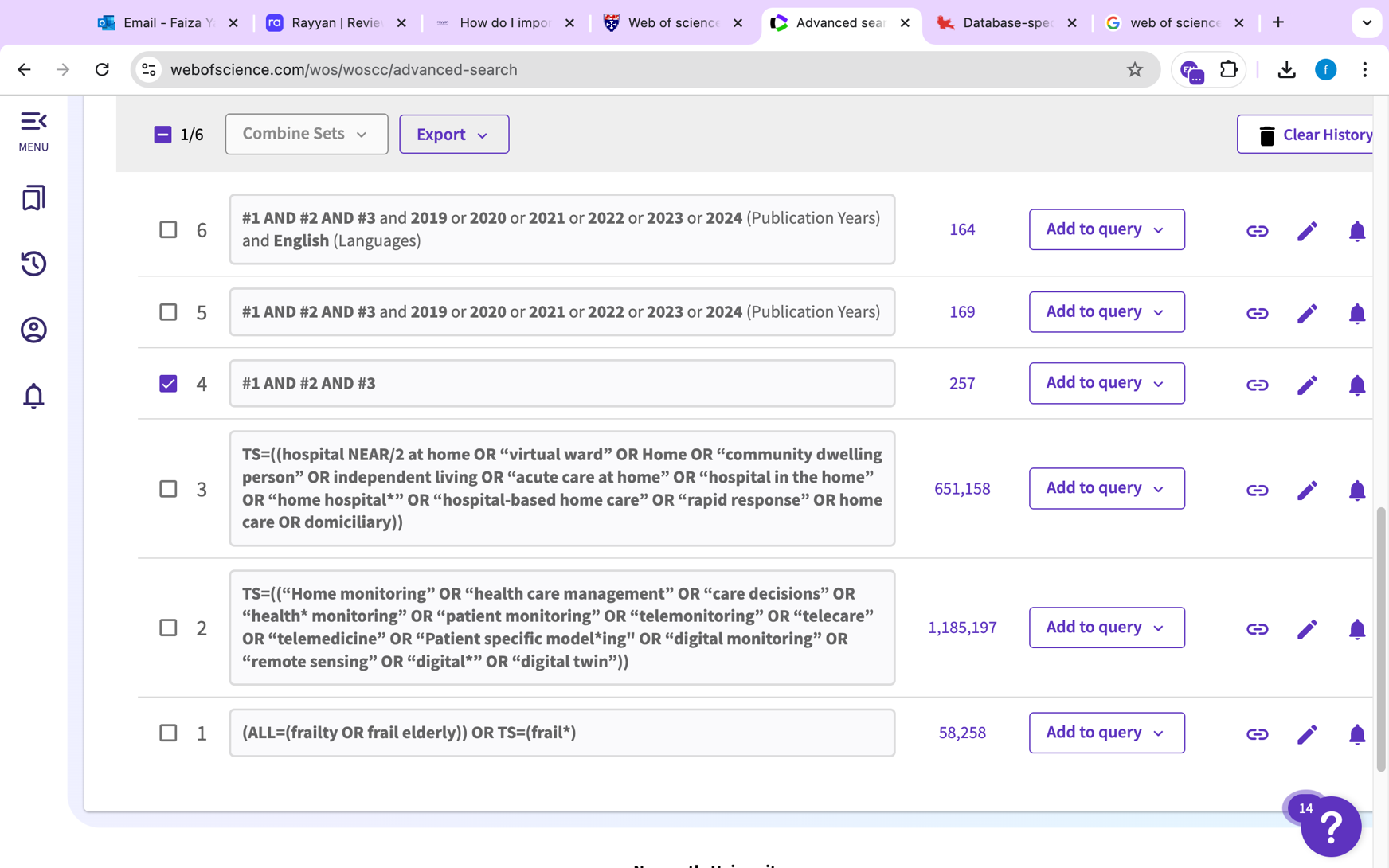
**

**Scopus**

( ( TITLE-ABS-KEY ( frail* ) OR ALL ( frailty ) OR ALL ( frail AND elderly ) ) ) AND ( TITLE-ABS-KEY ( "Home monitoring" OR "health care management" OR "care decisions" OR "health* monitoring" OR "patient monitoring" OR "telemonitoring" OR "telecare" OR "telemedicine" OR "Patient specific model*ing" OR "digital monitoring" OR "remote sensing" OR "digital*" OR "digital twin" ) ) AND ( TITLE-ABS-KEY ( hospital W/2 at AND home OR "virtual ward" OR home OR "community dwelling person" OR independent AND living OR "acute care at home" OR "hospital in the home" OR "home hospital*" OR "hospital-based home care" OR "rapid response" OR home AND care OR domiciliary ) ) AND PUBYEAR > 2018 AND PUBYEAR < 2025 AND ( LIMIT-TO ( LANGUAGE , "English" ) )

Results = **25**
