# Supplementary material for: Hospital at home digital twin for the management of patients with frailty: a scoping review protocol
Source: BMJ Open. 2025 Jun 17;15(6):e093418. doi: 10.1136/bmjopen-2024-093418 (PMC12182040; doi:10.1136/bmjopen-2024-093418)
Supplement: Supplementary file 4 [file bmjopen-2024-093418.reviewer_comments.pdf]

PEER REVIEW HISTORY

BMJ Open publishes all reviews undertaken for accepted manuscripts. Reviewers are asked to complete a checklist review form and are provided with free text boxes to elaborate on their assessment. These free text comments are reproduced below.

ARTICLE DETAILS

Title (Provisional)

Hospital at home digital twin for the management of patients with frailty: A scoping review protocol

Authors

Yahya, Faiza; Cooper, Matthew; Kassem, Mohamad; Nazar, Hamde

VERSION 1 - REVIEW

**Reviewer**	**1**
**Name**	**Malden, Stephen**
**Affiliation**	**The University of Edinburgh School of Health in Social Science, Scottish Collaboration for Public Health Research and Policy**
**Date**	**09-May-2025**
**COI**	**None**

Thank you for the opportunity to review this paper. This manuscript presents a protocol for a scoping review investigating the use of digital twin technologies to facilitate hospital at home care for people with frailty. The manuscript is well written, however currently lacks key details relating to evidence synthesis, and has only partially engaged with the most up to date literature on the topic and needs a stronger rationale for conducting a scoping review given recent existing reviews in the area. More details below:

Abstract:

A brief sentence in the abstract defining what an digital twin actually is would be helpful to the reader

Introduction:

Some brief presentation of the existing review literature in the digital twin field would further justify the rationale for this present scoping review. The majority of the existing literature in the area is cited later in the protocol, however this citation should be included: Machado, T.M. and Berssaneti, F.T., 2023. Literature review of digital twin in healthcare. Heliyon, 9(9).

Page 8 line 25: "no systematic reviews or scoping reviews were identified…" A systematic review of digital twins for home care was published in 2024 and is not cited in this

manuscript: Zafar, R.O., Rybarczyk, Y. and Borg, J., 2024. A Systematic Review of Digital Twin Technology for Home Care. ACM Transactions on Computing for Healthcare, 5(4), pp.1-24. A strong rationale should therefore be presented detailing what this scoping review will add to the literature in light of this. Suggest a detailed critique of this review is added where the gaps that the proposed scoping review will fill are made clear.

Methods:

The rationale for limiting searches to 2019 onwards makes sense, however providing a brief description of what the digital twin technology literature landscape looked like pre-covid would help further justify this. Suggest a brief summary in either the intro or discussion (wherever fits best) outlining the pre-covid literature, if it is not to be formally incorporated into the wider scoping review.

Search strategy databases:

Given the digital nature of the topic, I'd suggest searching ACM digital library and/or IEEE Xplore.

Analysis of evidence:

This section is currently too brief. Given the wide inclusion criteria relating to study design, a little more detail on evidence synthesis would be helpful here. I presume the qualitative content analysis referred to here would be for qualitative studies, while the tabulation and charting will cover quantitative results? This is a bit unclear at present. Generally, this section should include a clear description of what particular data will be extracted, and how it will be synthesised to answer the research questions and objectives. What specific aspects will be represented in tabular form? (e.g. population, setting, results summary, direction of intervention effect etc).

VERSION 1 - AUTHOR RESPONSE

**Reviewer's comments**<br>Reviewer: 1; Dr. Stephen Malden	**Author's response**
Thank you for the opportunity to review this paper. This manuscript presents a protocol for a scoping review investigating the use of digital twin technologies to facilitate hospital at home care for people with frailty. The manuscript is well written, however currently lacks key details relating to evidence synthesis, and has only partially engaged with the most up to date literature on the topic and needs a stronger rationale for conducting a scoping review given recent existing reviews in the area. More details below:	Thank you for your comments which we have addressed below:
**Abstract:**<br>A brief sentence in the abstract defining what a digital twin actually is would be helpful to the reader	Many thanks for highlighting that this clarity is needed. Clarification has now been added in lines 37-38.

**Introduction:**<br><br>Some brief presentation of the existing review literature in the digital twin field would further justify the rationale for this present scoping review. The majority of the existing literature in the area is cited later in the protocol, however this citation should be included: Machado, T.M. and Berssaneti, F.T., 2023. Literature review of digital twin in healthcare. Heliyon, 9(9).<br><br>**Page 8 line 25:** "no systematic reviews or scoping reviews were identified…" A systematic review of digital twins for home care was published in 2024 and is not cited in this manuscript: Zafar, R.O., Rybarczyk, Y. and Borg, J., 2024. A Systematic Review of Digital Twin Technology for Home Care. ACM Transactions on Computing for Healthcare, 5(4), pp.1-24. A strong rationale should therefore be presented detailing what this scoping review will add to the literature in light of this. Suggest a detailed critique of this review is added where the gaps that the proposed scoping review will fill are made clear.	Thank you for highlighting this. At the time of submission of this protocol, 9 months ago (Sept 2024), the systematic review suggested was not published.<br><br>We have however, considered both literature reviews for our full scoping review write-up.<br><br>We have updated the protocol based on your suggestions and agree that these are key papers which shape the trajectory of our research.<br><br>The reference by: Machado, T.M. and Berssaneti, F.T., 2023, has now been cited but has is largely bibliometric and does not capture the granularity required by our aims and objectives in this study. Page 7- Lines added 179-180.<br><br>Page 7 – Lines 177-178 has been updated to *"Furthermore, no systematic reviews or scoping reviews were identified to answer this research question in the context of HaH, and the evidence that exists is variable."*<br><br>Page 7- Lines 180-191 added with reference to [2].

**Methods:**<br><br>The rationale for limiting searches to 2019 onwards makes sense, however providing a brief description of what the digital twin technology literature landscape looked like pre-covid would help further justify this. Suggest a brief summary in either the intro or discussion (wherever fits best) outlining the pre-covid literature, if it is not to be formally incorporated into the wider scoping review.	Thank you for highlighting the need for clarification.<br><br>As highlighted by a recent systematic review [2], there is an absence of literature on Digital Twins in home care between 2014-2016. This is also corroborated with other literature [1] indicating the lack of DT literature prior to 2019, which emphasises the rationale for our approach.<br><br>A line has been added to the protocol and emphasised also in the full review.<br>See page 10; Lines 238-239 (methods) *"Additionally, recent studies have highlighted the lack of studies on DTs prior to 2019."*

**Search strategy databases:**<br>Given the digital nature of the topic, I'd suggest searching ACM digital library and/or IEEE Xplore.	Thank you for your useful and comprehensive comment. Please note that on conducting the search strategy we sought expertise from experienced research librarians and a professor of engineering with an extensive background in this field.<br>Preliminary searches on IEEE by the research team did not find any publications specific to HaH.<br><br>The search was designed to include Scopus database which indexes publications from both ACM and IEEE. Hence, the results of our search have already picked up sources from IEEE relevant to this study.

**Analysis of evidence:**<br>This section is currently too brief. Given the wide inclusion criteria relating to study design, a little more detail on evidence synthesis would be helpful here. I presume the qualitative content analysis referred to here would be for qualitative studies, while the tabulation and charting will cover quantitative results? This is a bit unclear at present. Generally, this section should include a clear description of what particular data will be extracted, and how it will be synthesised to answer the research questions and objectives. What specific aspects will be represented in tabular form? (e.g. population, setting, results summary, direction of intervention effect etc).	Thank you for highlighting this. Content analysis was used for qualitative evidence and where quantitative data was analysed by narrative synthesis. This has been updated in Lines 298-303.<br><br>Line 299-303 updated.<br>*"Results will be presented as a combination of tabular forms and charts depending on the nature of the results. Descriptive information (i.e. characteristics of the studies) will be presented in tabular form and other findings that answer the research questions will be presented as visual chart format that best represents the findings."*
Reviewer: 1<br>If you have selected 'Yes' above, please provide details of any competing interests.: Not applicable	No comment

References:

1. Machado, T.M. and Berssaneti, F.T., 2023. Literature review of digital twin in healthcare. Heliyon, 9(9).
2. Zafar, R.O., Rybarczyk, Y. and Borg, J., 2024. A Systematic Review of Digital Twin Technology for Home Care. ACM Transactions on Computing for Healthcare, 5(4), pp.1-24.
